# Perfluorinated compounds are related to breast cancer risk in greenlandic inuit: A case control study

**DOI:** 10.1186/1476-069X-10-88

**Published:** 2011-10-06

**Authors:** Eva C Bonefeld-Jorgensen, Manhai Long, Rossana Bossi, Pierre Ayotte, Gert Asmund, Tanja Krüger, Mandana Ghisari, Gert Mulvad, Peder Kern, Peter Nzulumiki, Eric Dewailly

**Affiliations:** 1 Centre for Arctic Environmental Medicine, Department of Public Health, Aarhus University, Denmark; 2 National Environmental Research Institute, Aarhus University, Denmark; 3 Institut National de Santé Publique du Québec, Québec, QC, Canada; 4 Dronning Ingrids Hospital, Nuuk Greenland

**Keywords:** PFCs, POPs, combined serum xenohormone and dioxin-like activities, n-3 fatty acids

## Abstract

**Background:**

Breast cancer (BC) is the most common cancer for women in the western world. From very few cases an extraordinary increase in BC was observed in the Inuit population of Greenland and Canada although still lower than in western populations. Previous data suggest that exposure to persistent organic pollutants (POPs) might contribute to the risk of BC. Rat studies showed that perfluorinated compounds (PFCs) cause significantly increase in mammary fibroadenomas. This study aimed at evaluating the association between serum levels of POPs/PFCs in Greenlandic Inuit BC cases and their controls, and whether the combined POP related effect on nuclear hormone receptors affect BC risk.

**Methods:**

Thirty-one BC cases and 115 controls were sampled during 2000-2003 from various Greenlandic districts. The serum levels of POPs, PFCs, some metals and the combined serum POP related effect on estrogen- (ER), androgen- (AR) and Ah-receptor (AhR) transactivity were determined. Independent student t-test was used to compare the differences and the odds ratios were estimated by unconditional logistic regression models.

**Results:**

We observed for the very first time a significant association between serum PFC levels and the risk of BC. The BC cases also showed a significantly higher concentration of polychlorinated biphenyls at the highest quartile. Also for the combined serum POP induced agonistic AR transactivity significant association to BC risk was found, and cases elicited a higher frequency of samples with significant POP related hormone-like agonistic ER transactivity. The AhR toxic equivalent was lowest in cases.

**Conclusions:**

The level of serum POPs, particularly PFCs, might be risk factors in the development of BC in Inuit. Hormone disruption by the combined serum POP related xenoestrogenic and xenoandrogenic activities may contribute to the risk of developing breast cancer in Inuit. Further investigations are needed to document these study conclusions.

## Background

Breast cancer (BC) is the most common cancer for women in the western world and the incidence has been increasing since 1940. The highest incidence rates are observed in North America, and the lowest risk is found in Asia and Africa [1]. Breast cancer is also the most common cancer in females in Europe with the highest incidence in The Netherlands and Denmark and lowest in the eastern part of Europe [2]. From very few cases in the 1970's an extraordinary increase in BC has been observed in the Inuit population of Greenland and Canada today [3,4]. Known established breast cancer risk factors include genetic inheritance e.g. mutations in the BRCA-1 and BRCA-2 genes [5], lifelong exposure to estrogens (early menarche and late menopause increases the risk), obesity after menopause, alcohol, smoking and high intake of fat [2,6,7]. Some factors seem to reduce the risk such as low age at first birth, large number of full term pregnancies and long duration of breastfeeding [6,7]. Thus, BC risk is influenced by genetics and reproductive history, but the known risk factors only explain less than a third of all cases and more than 70% of women diagnosed with BC have no inherited or sporadic cancer. The risk of BC is thought to be modified by lifestyle and environmental exposures. Results from many studies have confirmed that BC is not a single disease with variable morphologic features and biomarkers, but rather a group of molecularly distinct neoplastic disorders [8].

The susceptibility to BC upon environmental exposures might not only be as child, in puberty or young but can be pre-determined in utero through alterations of the hormonal environment caused by either maternal diet and/or exposure to environmental chemicals with endocrine activities that can modify the epigenome. These epigenetic modifications, inherited by somatic daughter cells, lead to changes in mammary gland development and increase the vulnerability for malignant transformation [9]. An array of legacy persistent organic pollutants (POPs) including polychlorinated dibenzodioxins and polychlorinated dibenzofurans (PCDD/PCDF), polychlorinated biphenyls (PCBs), and organochlorine pesticides (OCPs) are potential endocrine disrupters and can play an important role in the risk of BC. These persistent and lipophilic compounds do biomagnify through the food web and bioaccumulate age dependently in human and animals. PCBs have been associated with effects being relevant for development of BC such as estrogenic, tumour promoting, and immunosuppressive activities [10]. Evidence regarding organochlorine exposure and BC risk is controversial. A case-control study of Danish women showed a significant doses relation between the risk of BC and the serum level of the pesticide dieldrin, and a non-significant relation of BC risk at the highest level of dieldrin and PCBs among BC women with a tumour with mutant p53 [11,12]. In contrast, Raaschou et al. 2005 found no association between persistent organochlorines (14 pesticides and 18 PCBs) levels in adipose tissues and breast cancer in postmenopausal Danish women [13]. Thus, no strong evidence for the role of organochlorines, including PCBs, in BC development was found. However, two studies found a 3-fold risk in postmenopausal breast cancer with a A2455G mutation in the P450 polymorphic CYP1A1 gene and high PCB levels compared with wild-type alleles and low PCBs [14]. These associations between PCB exposure and BC risk among genetically susceptible and racially subgroups warrant further studies.

Perfluorinated compounds (PFCs) are a large group of chemicals used since the 1950's in different industrial and commercial applications (e.g. Teflon, carpets, furniture, food stuff packing etc.). For a long time, these fluorinated chemicals were considered metabolically inert and nontoxic [15] and the carbon-fluorine bond renders theses chemicals very resistant to biodegradation and therefore persistent in the environment [15]. Available evidence suggest the transformation or biodegradation of precursor perfluorinated chemicals occurs by both abiotic and biotic degradation pathways where perfluorooctane sulfonate (PFOS) and perfluorooctanoic acid (PFOA) are typical final degradation products [16,17]. In 2001, it was discovered that the PFCs were accumulating in the environment and human tissues with a global distribution [16-18]. Unlike the legacy POPs (e.g. PCBs, OCPs) - which accumulate in lipid rich tissues - PFCs bind to blood proteins and accumulate mainly in liver, kidney and bile secrets [19]. Humans are exposed to PFCs through occupational settings, environmental exposures and/or through contact with consumer goods (diet, air, water, food and household dust) where PFCs have been found.

The perfluoroalkyl acids (PFAAs) include the perfluorocarboxylated acids (PFCAs) and perfluorosulfonated acids (PFSAs), and the PFAAs include the two most studied PFCs: PFOS and PFOA. These two compounds are the most studied because existing laboratory procedures in the past did not allow analyses of other PFCs that in general exist in lower concentrations. PFOA and PFOS are persistent in the environment and found in human blood, breast milk and liver with half lives of 4 to 10 years [20]. The PFCs are found globally and governmental regulations in USA and Europe on use and production of specific compounds such as PFOS and PFOA have been made. Recently, PFOS has been added to Annex B of the Stockholm Convention on POPs [21].

Biomonitoring studies have been carried out in almost all part of the world in order to assess PFAAs levels and temporal trends in the general population [22,23]. European studies observed serum and plasma concentrations range from 1 to 116 ng/ml for PFOS and from 0.5 to 40 ng/ml for PFOA. The average plasma levels of PFOS and PFOA in Danish pregnant women was 35.3 and 5.6 ng/ml, respectively, similar to most levels reported for western country populations during the same decade [24]. For middle aged women in Norway slightly lower levels were reported (medians: PFOS; 20 ng/ml; PFOA; 4.4 ng/ml). Mean and median concentrations from North American populations appear to be slightly higher than European, Asian and Australian populations [22]. Recently we performed a survey of serum PFCs in Greenlandic Inuit [25] showing similar PFOS levels in Inuit women as found for Danish and European women but lower PFOA levels.

The maternal level of PFOA or PFOS was found associated with various reproductive and child health outcomes [24,26-29], and seems to impact maternal fecundity [27].

The biological effects of PFCs have been studied in more detail mainly in rodents; little data are available for other species and humans [19,30]. Studies in animals have documented an array of toxicological outcomes including liver hypertrophy and tumours [31], thyroid hormone alterations, decreased serum cholesterol and glucose, developmental toxicity, immunotoxicity, and carcinogenic potency [32,33]. Animal and *in vitro *studies have also suggested that PFAAs may have potential geno- and neurotoxic effects [34-36]. U.S. EPA has proposed PFOA to be deemed as a rodent carcinogen with relevance to humans [37].

A 2-year study in rats [38] reported a statistically significant increase in mammary fibroadenomas and Leydig cell adenomas suggesting impact of PFOA on reproductive tissues. Because of these data the U.S. EPA Science Advisory Board recommended to reconsider the possible impact of PFOA on mammary tissues. In 2007 White and coworkers reported [39] that gestational exposure to PFOA in mice compared to non exposed controls was associated with altered mammary gland development in dams and female offspring. A significant reduction in mammary differentiation among exposed dams was evident, and also affected the epithelial involution and altered milk protein gene expression [39,40].

Recently, estrogen-like properties of PFCs were reported in human MCF-7 breast cancer cells suggesting endocrine potentials [41].

The objective of this BC case-control epidemiologic study in Greenlandic Inuit women was to evaluate the serum level of legacy POPs and PFCs, blood metals, and the combined xenobiotic serum POP related effect on the functions of the estrogen- (ER) and androgen receptors (AR) and the aryl hydrocarbon receptor (AhR) functions.

## Methods

### Study population and Data collection

**Figure 1:**
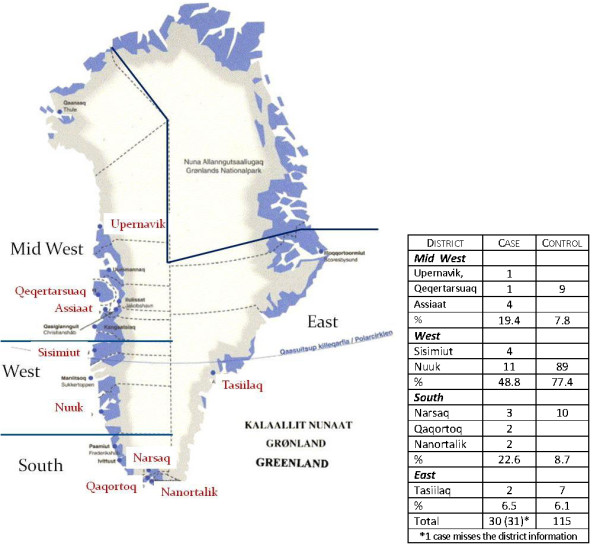
**Geographical distribution of the breast cancer cases and controls**.

The subjects of breast cancer cases were taken from Inuit women at the "Dronning Ingrids Hospital" in Nuuk, where all breast cancer cases in Greenland are registered. Approximately 80% of all BC cases were included in the sampling period 2000-2003. Subjects of controls were selected by frequency matching age and districts from a cross sectional study of POP concentrations and bone ultrasound measurement [42] and the Arctic Monitoring and Assessment Programme (AMAP) study [43]. The controls were matched with the cases having similar frequency of age ≤ 50, 51-55, 56-59, ≥ 60yrs (see table 1) and then frequency matched with the cases of the districts (see table of Figure 1). All subjects, 31 cases and 115 controls, were of Greenland Inuit decent, defined as having more than two grandparents born in Greenland. The sampling period was 2000-2003 with subjects from Nuuk, Upernavik, Qeqertarsuaq, Narsaq, Tasiilaq, Qaqortoq, Sisimiut, Assiaat, and Nanortalik. Figure 1 shows a map of Greenland, with the collection sites.

**Table 1:** Demographic, lifestyle and reproductive characteristics of breast cancer patients and controls

Parameters	Cases				Controls				p value
	
	N (n)	median	95% CI	Min-max	N (n)	median	95% CI	Min-max	
**Demographic and lifestyle factors**									

Age (years)	31 (31)	50	46.1; 56.7	29.0-80.0	115 (115)	54	49.1; 53.5	18-66	0.34

≤ 50	17 (54.8%)				37 (32.2%)				

51-55	3 (9.70%)				26 (22.6%)				

56-59	3 (9.70%)				17 (14.8%)				

≥ 60	8 (25.8%)				35 (30.4%)				

BMI (kg/m^2^)	31 (13)	26.9	24.0; 29.1	16.5-34.4	115 (115)	26.4	26.4;27.2	16.6-43.4	0.69

*<25*	*4*	*23.5*	*16.0; 22.3*	*16.5-25.0*	*42*	*23.0*	*21.7;23.0*	*16.1-25.0*	*0.84*

*25-29*	*8*	*27.6*	*26.6; 28.9*	*25.8-29.6*	*42*	*27.6*	*26.9;27.8*	*25.1-19.9*	*0.46*

*> 30*	*1*	*34.4*	*-*	*-*	*31*	*33.2*	*33.1; 35.9*	*30.3-43.4*	*-*

n-3/n-6	31 (29)	0.5	0.4; 0.6	0.2-1.7	115 (115)	0.5	0.5; 0.7	0.1-2.2	0.31

Serum cotinine (ng/ml)	31 (28)	11.6	33.3; 140.0	0-600.0	115 (96)	120	121.0; 188.0	0.0-799.0	**0.052**

Smoking status	31 (26)				115 (115)				0.77

*Never*	5 (19.2%)				21 (18.3%)				

*Former*	3(11.5%)				20 (17.4%)				

*Current*	18 (69.2%)				74 (64.3%)				

**Reproductive factors**									

Total number of full term pregnancies	31(16)	2.0	1.7; 2.8	1-4	115 (89)	3.0	3.2; 4.1	0.0-11.0	**<0.0001**

Ever breastfed	31(17)				115 (85)				

Yes (%)	15 (88.2%)				76 (89.4%)				0.89

Menopausal status	31 (20)				115 (89)				

Premenopausal (%)	11 (55.0%)				16 (18.0%)				**0.001**

Postmenopausal (%)	9 (45.0%)				73 (82.0%)				**0.001**

Serum E2 (nmol/l)	31(24)*	0.09	0.09; 0.23	0.03-0.51	115(59)	0.07	0.05; 0.16	0.01-1.59	0.10

Premenopausal	8	0.24	0.12; 0.38	0.06-0.44	11	0.13	0.05; 0.55	0.03-1.59	

Postmenopausal	8	0.07	0.01; 0.25	0.03-0.51	48	0.07	0.06; 0.08	0.01-0.17	

N: total number of subjects, n: number of subjects having information for the corresponding parameters.

BMI: body mass index

*: 24 cases had E2 data but for 8 no menopause status information was given.

All subjects completed the validated Danish standard questionnaire. It included questions about demographic and lifestyle parameters and allowed to document the following risk factors for breast cancer: age, breastfeeding, Body Mass Index (BMI), smoking, menopause status.

### Blood sample collection and analyses

Blood samples were taken when the breast cancer was diagnosed and for controls when enrolled in the study following the standard procedure and stored at -80°C until analyses.

Concerning the chemical analyses and bio-activity measurements both cases and controls were batch together in the run of assays and the persons running the assays were blinded to the samples of cases and controls

### Plasma legacy POPs and plasma lipid

The lipophilic plasma legacy POPs and lipids were analysed at the Centre de Toxicologie of the Institut National de Sante Publique du Quebec (Quebec, Canada) (CTQ), a certificated laboratory by Canadian Association for Environmental Analytical Laboratories. Plasma was extracted by 1:1:3 mixture of ammonium sulphate: ethanol: hexane and then concentrated and purified on two florosil columns. Twelve PCB congeners [International Union for Pure and Applied Chemistry (IUPAC) no. 99, 101, 105, 118, 128, 138, 153, 156, 170, 180, 183, 187], and eight OCPs [p,p'-DDT(dichlorodiphenyltrichloroethane) and its major metabolite p,p'-DDE (p,p'-dichlorodiphenyl-dichloroethylene), β-hexachlorocyclohexane (β-HCH), aldrin, hexachlorobenze (HCB), oxychlorodane, cis-nonachlor and trans-nonachlor] were analysed in purified extracts by high-resolution gas chromatography with electron capture detection. The detection limit was 0.08 μg/L for p, p'-DDE, p, p'-DDT and β-HCH, and 0.04 μg/L for other pesticides and PCBs [42]. The measured PCBs and OCPs were grouped as sum PCB and sum OCPs.

Plasma lipids were measured at CTQ using standard enzymatic procedure. The total plasma lipid concentration was obtained from cholesterol esters, free cholesterol, triglycerides and phospholipids as described previously [44]. To obtain the lipid adjusted POPs data (μg/kg lipid), the measured PCBs and pesticides (μg/L) were divided by total plasma lipid (g/L) and multiplied by 1000.

### Serum perfluorinated compounds (PFCs)

Serum PFCs were measured at the National Environmental Research Institute, Aarhus University, Denmark. Perfluoroheptanoic acid (PFHpA), perfluorooctanoic acid (PFOA), perfluorononanoic acid (PFNA), perfluorodecanoic acid (PFDA), perfluoroundecanoic acid (PFUnA), perfluorododecanoic acid (PFDoA), perfluorotridecanoic acid (PFTrA), perfluorohexane sulfonate (PFHxS), perfluorooctane sulfonate (PFOS) and perfluorooctane sulfonamide (PFOSA) were measured in serum extracts. The extraction method was based on ion pairing as described previously [45]. Instrumental analysis was performed by liquid chromatography-tandem mass spectrometry (LC-MS-MS) with electrospray ionization (ESI). The analytes were separated on a C18 Betasil column (2.1 × 50 mm, Thermo Hypesil-Keystone, Bellafonte, PA, USA) using an Agilent 1100 Series HPLC (Agilent Technologies, Palo Alto, CA). The HPLC was interfaced to a triple quadruple API 2000 (Sciex, Concorde, Ontario, Canada) equipped with a TurboIon Spray™ source operating in negative ion mode. Detection of the analytes was based on retention time and the most abundant mass transition corresponding to an authentic standard. Confirmation of analyte identity was based on the relative response of the secondary mass transition to the primary mass transition. Quantification of the analytes was done using response factors calculated from a four point calibration curve consisting of blank samples (rabbit serum) spiked with the analytes in the concentration range 1-50 ng/ml and extracted following the same procedure as samples.

The samples were extracted and analyzed in batches together with a procedural blank. The target compounds were not detected in any of the blank samples. The detection limit of the analytical method (MDL) was defined as those concentrations of the analytes needed to produce a signal-to-noise ratio (S/N) of 3:1. Detection limits ranged from 0.1 to 0.4 ng/ml. Method performance is currently tested by participating in the AMAP Ring Test for Organic Pollutants in Human Serum organized by Institute Nationale de santé publique du Québec [46]. Satisfactory Z-scores were obtained by our laboratory in the tests for PFCs.

The analysed PFCs were grouped into sumPFSA (sum of PFOS, PFHxS and PFOSA) and sumPFCA (sum of PFHpA, PFOA, PFNA, PFDA, PFUnA, PFDoA and PFTrA).

### Blood metals

Whole blood metals including selenium (Se), zinc (Zn) and the heavy metals lead (Pb), mercury (Hg) and cadmium (Cd) were measured using Inductively Coupled Plasma Mass Spectrometry (ICP-MS) after digesting blood with nitric acid in the microwave at the National Environmental Research Institute, Aarhus University, Denmark.

### Plasma fatty acids

Plasma fatty acids were determined by capillary gas-liquid chromatography at the Biology Department, University of Guelph, Canada [47]. The fatty acids composition of plasma phospholipids was expressed as a percentage of the total area of all fatty acid peaks from 14:0 to 24:0. Plasma phospholipids of fatty acids correspond to relative percentages of total fatty acids by weight. The n-3 polyunsaturated fatty acids were reported on the sum of C18:3, n-3; C18:4, n-3; C20:3, n-3; C20:4, n-3; C20:5, n-3; C22:5, n-3; C22:6, n-3, and the n-6 fatty acid acids was the sum of C18:2, n-6; C18:3, n-6; C20:2, n-6; C20:3, n-6; C20:4, n-6; C22:2, n-6; C22:4, n-6 and C22:5, n-6. The ratio between n-3 and n-6 is known to be a strong indicator of marine food intake and thus a good indicator of the relative consumption of traditional versus imported food [48,49].

### Serum cotinine determination

Cotinine is a metabolite of nicotine. The level of cotinine in the blood is proportionate to the amount of exposure to tobacco smoke and thus it is a valuable indicator of tobacco smoke exposure. The Calbiotech Cotinine Direct ELISA Kit was used to measure the serum cotinine (Calbiotech Inc., CA, USA) at Centre of Arctic Environmental Medicine & Cellular & Molecular Toxicology,, Department of Public Health, Aarhus University, Denmark. This assay is a solid phase competitive ELISA and the absorbance was read on ELISA Reader at 450 nm. The serum cotinine concentration was expressed as ng/ml and the limit detection was 1 ng/ml.

### Measurement of serum estradiol

Solid phase fluoronimmunoassay was employed to determine serum 17 β-Estradiol using DELFIA^® ^Estradiol kit (PerkinElmer Life and Analytical Sciences, Wallac Oy, Turku, Finland) at Centre of Arctic Environmental Medicine & Cellular & Molecular Toxicology, Department of Public Health, University of Aarhus, Denmark. The principle of this assay is based on competition between europium-labelled estradiol and sample estradiol for polyclonal anti-estradiol antibodies. The fluorescence of the samples were measured on a fluorometer (Wallac 1420 Multilabel Counter, Perkin Elmer life science, FIN) with protocol "56 E2" for automatic measurement and result calculation. The serum E2 concentration was expressed as nmol/L. The sensitivity of the assay was 0.05 nmol/L, and the intra- and inter-assay coefficient of variation were less than 10%.

### Serum POP related xenobiotic induced receptor transactivities

SPE-HPLC fractionation of the serum samples for determination of ER and AR transactivity.

For determination of the combined serum POP related xenoestrogen (XER) and xenoandrogen (XAR) receptor transactivity a Solid Phase Extraction (SPE) and a High-Performance Liquid Chromatography (HPLC) fractionation was performed on 3.6 ml serum to obtain the serum fraction (F1) containing the actual mixture of bio-accumulated lipophilic POPs separated from endogenous hormones. The SPE-HPLC F1 extracts were analyzed for the actual combined xenohormone activity in the ER and AR transactivation assays [50-52].

### Measurement of XER-transactivity and XAR-transactivity

Determination of the ER transactivation was carried out in the stable transfected MVLN cells, carrying the ERE-luciferase reporter vector (kindly provided by M. Pons, France) [51,53].

The AR transactivation was determined in the Chinese Hamster Ovary cells (CHO-K1) by transient co-transfection with the MMTV-LUC reporter vector (kindly provided by Dr. Ronald M. Evans, Howard Huges Medical institute, CA) and the AR expression plasmid pSVAR0 (kindly provided by Dr. A.O. Brinkmann, Erasmus University, Rotterdam) [52]. The luciferase activity was measured in a LUMIstar luminometer (BMG Lumistar, RAMCON, Denmark) and corrected for cell protein by fluorometric measurements in the WALLAC VICTOR2 (PerkinElmer, USA) at 355/460 nm wavelength as described [53-55].

In each assay all samples were tested in triplicate in two sets of tests: 1) the effect of the serum F1 POP extract only (termed XER/XAR) to test primarily for agonistic effect. The response of the serum F1 POP extract was compared to the reference solvent control of the analyses. When the F1 POP extract was significantly higher compared to that of solvent control (p <0.05), it was termed as significantly agonistic XER/XAR (i.e. significantly increased xenoestrogenic/xenoandrogenic transactivity); 2) the competitive xenohormone transactivity was determined upon co-treatment with 17 β-estradiol (E2) at EC_40_-E2 or the synthetic testosterone R1881 at EC_50_-R1881 and the serum F1 POP extract (termed XERcomp/XARcomp) to test primarily for antagonistic effects on ligand induced receptor transactivity (i.e. response of serum F1 POP extract plus E2 or R1881 were significantly lower than the response of E2 or R1881 alone), but if the response was significantly higher than the ligand reference values an additive or synergistic effect is indicated [51,52]. Measurements of the serum xeno-estrogen and xeno-androgen receptor transactivity were conducted at Centre of Arctic Environmental Medicine & Cellular & Molecular Toxicology, Department of Public Health, Aarhus University, Denmark.

### Serum extraction of POPs for AhR-transactivity determination

A semi-automated solid-phase extraction method was used to prepare purified extracts for AhR-mediated transcriptional activity analyses from a single 5-ml plasma sample as described in Medehouenou et al. [54]. In short, the plasma sample was mixed with equal parts of formic acid and deionised water and extracted using a methanol: dichloromethane mixture (1/9). Then the extract was cleaned up on activated silica/acidic silica column and the compounds were eluted with dichloromethane. The solvent was evaporated to dryness and the resulting fraction reconstituted in 5 μl of dimethylsulfoxide for the measurement of AhR-mediated transcriptional activity [54].

### Measurement of AhR-mediated transactivity

Detail procedure of AhR-mediated transactivity measurement was described elsewhere [54,55]. In short, the bioassay used to measure the AhR mediated transactivity was based on the expression of the firefly luciferase in H4IIE.Luc cells resulting from the activation of the AhR pathway by AhR activating compounds. H4IIE.Luc cells (kindly donated by A. Brouwer, BioDetection Systems B.V., Amsterdam, The Netherlands) were obtained by transfecting rat hepatoma H4IIE cells with the luciferase reporter gene plasmid pGudLuc1.1 [56,57]. After H4IIE.Luc cells were exposed to TCDD standards and the cleaned plasma extracts for 24 h, the luciferase activity was measured. AhR-mediated transcriptional activity of cleaned plasma extracts was interpolated onto the TCDD dose-response curve of AhR mediated luciferase activity and expressed as TCDD equivalents (AhR-TEQ), which represents the total TCDD-toxic potency of a mixture of dioxin-like compounds. The limit of detection was 30 pg TEQ/L, corresponding to approximately 5 pg TEQ/g lipids [54]. Measurement of AhR-mediated transactivity was performed at the Centre de Toxicologie (CTQ) of the Institut National de Sante Publique du Quebec (Quebec, Canada).

### Statistical analysis

The distribution of data was checked by Q-Q plots. The natural logarithmic transformed variables improved the normality and homogeneity of variance and thus the comparison analysis was performed on the ln-transformed data. Independent student t-test was used to compare the graphical variables (age, BMI, fatty acids), chemical variables (PFCs, POPs, metals), xenobiotic induced transactivities (XER/XERcomp, XAR/XARcomp, AhR-TEQ) between cases and controls. Comparison of chemical variables and xenobiotic induced receptor transctivities was also performed by adjusting for age, BMI, number of pregnancies and smoking (serum cotinine) using ANCOVA analysis. Pearson's chi-square test was used to check the difference between cases and controls for frequency of breastfeeding, menopausal status and agonistic XER/XAR.

Unconditional logistic regression models were used to estimate the odds ratios (ORs) and 95% confidence intervals (95% CI) under controlling for potential confounders. Potential confounders considered for this analysis included age, BMI, total number of full-term pregnancies, breastfeeding, menopausal status and serum cotinine based on a priori consideration of the research design and well-established breast cancer risk factors. Each potential confounder was included in the model one by one with the chemical variables or xenobiotic induced receptor transactivities and compared to the model only with the chemical measurements or xenobiotic induced receptor transactivities. The confounder was identified when the difference of beta coefficients was more than 15%.

All statistical analysis was performed using SPSS version 17.0 (SPSS Inc. Chicago, IL, USA) conducted at Centre of Arctic Environmental Medicine & Cellular & Molecular Toxicology,, Department of Public Health, Aarhus University, Denmark. The statistical significant level was set to p ≤ 0.05.

## Results

### Demographic, lifestyle and reproductive characteristics of the study population

Table 1 summarize the demographic, lifestyle and reproductive characteristics of the breast cancer (BC) cases and controls. Median age of the BC patients and controls was 50 and 54 years, respectively, and no significant age difference was observed. The age distributions at ≤ 55, 56-59 and ≥ 60 of cases and controls were similar. Cases and controls had similar BMI. The seafood intake, represented by the ratio of n-3 fatty acid to n-6 fatty acid (n-3/n-6), did not differ between cases and controls. To evaluate the current smoking status of the participants, serum cotinine levels of participants were measured, and borderline lower level was found for BC cases compared to their controls, indicating less current smoking among the cases than control participants. For the smoking behaviour obtained from the questionnaire, no significant difference between cases and controls was observed (Table 1).

As shown in Table 1, the BC cases had lower full pregnancies numbers compared to the controls. However, this difference might be suggestive since only 52% of cases and 77% of controls had information on pregnancy; the proportion of breast feeding of BC patients was similar to that of the control participants. Similar percent of information on menopausal status for cases and controls (65% and 77%, respectively) was given. A substantial proportion of breast cancer patients were premenopausal (55%) while most of controls were postmenopausal (82%). The serum level of estradiol (E2) was non-significantly higher in BC cases compared with controls. In both cases and controls the premenopausal women had higher E2 level (Table 1).

### Serum levels of POPs, metals and xenobiotic induced receptor transactivities POPs

The serum level of perfluorinated compounds (PFCs) was clearly significant higher in BC cases than controls (Table 2), and this significant difference persisted upon adjustment for age, BMI, pregnancy and cotinine. For PFOS and the sum of perfluorsulfonated acids (sumPFSA), breast cancer patients had double median level compared to the controls (p < 0.0001, Table 2), and for PFOA and sum PFCA the significant difference between cases and controls was given by p = 0.04 and p = 0.001, respectively. No significant difference was observed between cases and controls for the legacy POPs such as PCBs and OCPs (Table 2). However, when the PCBs were subdivided into quartiles, the highest PCBs quartile (PCBs > 2645 ug/kg lipid) was significantly higher for the cases compared with the controls (p = 0.02, Table 2). Also upon pooling the data for legacy POPs and PFCs, the cases had significantly higher level than that of controls (p = 0.007, Table 2). Similar tendency was observed when data was stratified by menopausal status (see additional file 1).

**Table 2:** Serum levels of POPs and blood metals in breast cancer patients and controls

	Cases				Controls				p value
	
	N (n)	median	95% CI	Min-max	N (n)	median	95% CI	Min-max	
**POPs**									

PFOS(ng/ml)	31(31)	45.6	45.7; 69.3	11.6-124	115(98)	21.9	31.1; 46.0	1.5-172	**<0.0001**

PFOA(ng/ml)	31(31)	2.5	2.2; 3.4	0.2-7.2	115(98)	1.6	2.11; 2.9	0.2-7. 6	**0.04**

Sum PFSA(ng/ml)	31(31)	48.2	49.6; 74.6	13.2-133	115(98)	24.6	33.7; 49.7	2.0-184	**<0.0001**

Sum PFCA(ng/ml)	31(31)	8.0	7.9; 11.9	3.3-21.4	115(98)	5.2	6.4; 9.2	1.0-28.1	**0.001**

Sum PCB(μg/kg lipid)	31(30)	2049	1596; 2870	150.5-6528	115(115)	1867	1759; 2203	92.7-5640	0.69

*< 920 (μg/kg lipid)*	*10*	*548*	*386; 734*	*151-857*	*23*	*451*	*379; 581*	*92.7-893*	*0.41*

*920-1745(μg/kg lipid)*	*4*	*1343*	*806; 1907*	*1016-1724*	*31*	*1314*	*1263; 1438*	*1015-1737*	*0.95*

*1745-2645(μg/kg lipid)*	*7*	*2222*	*2088; 2499*	*1995-2585*	*32*	*2163*	*2118; 2315*	*1753-2623*	*0.46*

*> 2645(μg/kg lipid)*	*9*	*4248*	*3637;5231*	*2923-6528*	*29*	*3308*	*3267; 3903*	*2709-5640*	***0.02***

Sum OCP (μg/kg lipid)	31(30)	2532	2224; 3581	250-7320	115(115)	2400	2130; 2678	128-8804	0.19

Sum DL-PCB (μg/kg lipid)	31(30)	149	141; 253	17.9-527	115(115)	198	189; 238	8.4-596.0	0.39

Sum PCB + sum OCP (μg/kg lipid)	31(30)	4424	3861; 6410	401-11849	115(115)	4206	3902; 4869	236-13300	0.35

Sum PCB + sum OCP + sum PFSA + sum PFCA (ng/ml)	31(31)	81.0	83.1; 124	22.1-231.8	115(115)	59.4	66.6; 88.0	4.9-298.0	**0.007**

**Metals**									

Se (μg/kg)	31(30)	216	195; 439	101-1877	115(115)	265	294; 405	86.6-1805	0.56

Cd (μg/kg)	31(30)	0.9	0.6; 1.1	0.0-2.7	115(111)	1.03	1.11; 1.51	0.0-6.5	0.09

Hg (μg/kg)	31(30)	16.9	14.4; 24.6	1.54-61.7	115(115)	14.6	15.3; 23.8	0.4-194.0	0.30

Pb (μg/kg)	31(30)	42.7	37.1; 60.4	10.8-134	115(115)	53.2	60.6; 86.6	2.1-499.0	0.23

Zn (μg/kg)	31(30)	5643	5360; 6270	3013-8583	115(89)	4879	4754; 5301	2431-10822	**0.003**

N: total number of subjects, n: number of subjects having information for the corresponding parameters.

CI: confidence interval. SumPFSA: sum of PFOS, PFHxS and PFOSA; sumPFCA: sum of PFHpA, PFOA, PFNA, PFDA, PFUnA, PFDoA and PFTrA; sum PCB: sum of PCB 99, 101,105,118,128,138,153,156,170,180,183,187; sumOCP: sum of p, p'-DDT, p, p'-DDE, β-HCH, aldrin, HCB, oxychlorodane, cis-nonachlor and trans-nonachlor. SumDL-PCB (dioxin-like PCB): sum of PCB 105, PCB 118, and PCB 156.

### Metals

Levels of selected trace elements and heavy metals in the study participants are given in Table 2. The level of Zn was significantly higher in cases, and a borderline higher level of Cd was found for controls. Although not significant the selenium (Se) level in BC cases tended to be lower than that of controls. But for the heavy metals Pb and Hg, no significant difference was observed between cases and controls.

### POP related receptor transactivities

**Figure 2:**
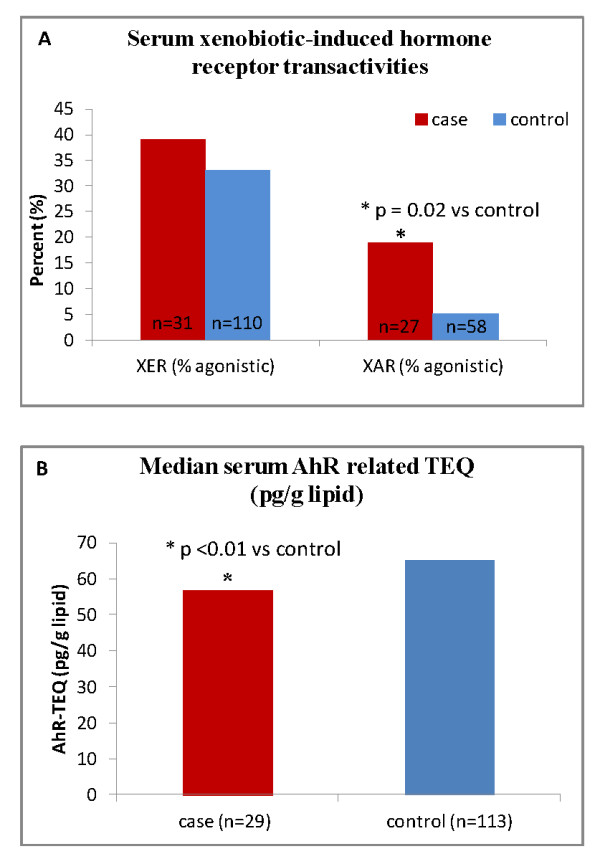
**Levels of serum POP related xenobiotic induced transactivities of breast cancer cases and controls**. A) serum xenobiotic agonistic induced ER and AR receptor transactivity; B) serum xenobiotic induced AhR transactivity (see legend to Table 3). % agonistic XER and XAR indicate a significant increase for the XER and XAR transactivity compared to the the solvent control. AhR-TEQ: AhR-TCDD equivalent. *: significant differences between cases and controls.

The xenobiotic potential of the extracted serum POP mixture was analyzed for effects on nuclear receptor transactivities such as ER, AR and AhR *ex vivo *upon exposure of the respective cell cultures as shown in Table 3. Serum xenoestrogenic transactivities (XER, XERcomp) did not differ significantly between the two groups, but BC cases elicited a higher subject sample frequency of significant agonistic xenoestrogenic transactivity compared to controls (38.7% vs. 32.7%; Table 3 and Figure 2). The agonistic xenoandrogenic transactivity (XAR) of cases was significantly higher than that of controls (p = 0.01, Table 3), and the cases also elicited higher subject frequency of significantly increased agonistic XAR (18.5% vs. 5.2%; Table 3 and Figure 2) compared to the controls. For the XER and XAR data no differences was found before and upon adjustment for the confounder's age, BMI, pregnancy and cotinine. Moreover, the serum AhR toxic equivalent (AhR-TEQ) of cases was lower than controls (p = 0.009, Table 3 and Figure 2); however, upon adjustment for age, BMI, pregnancy and cotinine the significance disappeared (data not shown).

**Table 3:** Serum POP related xenobiotic induced receptor transactivities in breast cancer patients and controls

Parameters	Cases				Controls				p value
	
	N (n)	median	95% CI	Min-max	N(n)	median	95% CI	Min-max	
**XER **(RLU/μg protein)	31(31)	1.07	1.02; 1.10	0.80-1.30	115(110)	1.08	1.05; 1.11	0.54-1.34	0.69

% agonistic XER		38.7%				32.7%			0.54

% downregulated XER		9.7%				8.2%			0.79

**XERcomp **(RLU/μg protein)	31(31)	1.06	1.00; 1.08	0.79-1.27	115(110)	1.04	1.00; 1.05	0.55-1.26	0.58

% additive XERcomp		22.6%				19.1%			0.67

% antagonistic XERcomp		12.9%				12.7%			0.16

**XAR **(RLU/μg protein)	31(27)	1.02	0.97; 1.25	0.56-2.01	115(58)	0.91	0.86; 0.99	0.40-1.74	**0.01**

% agonistic XAR		18.5%				5.17%			**0.02**

% downregulated XAR		11.1%				24.1%			0.17

**XARcomp **(RLU/μg protein)	31(27)	0.69	0.61; 0.82	0.14-1.33	115(58)	0.72	0.68; 0.79	0.29-1.23	0.51

% additive XARcomp		3.7%				0.0			0.14

% antagonistic XARcomp		55. 6%				36.2%			0.59

**AhR-TEQ **(pg/L)	31(29)	292	285; 568	31.41-1442	115(113)	503	771; 1544	112-12757	**<0.0001**

**AhR -TEQ **(pg/g lipid)	31(29)	56.8	46.40; 90.80	8.97-231	115(113)	65.4	103; 219	11.50-2086	**0.009**

N: total number of subjects, n: number of subjects having information for the corresponding parameters.

XER and XAR: ER and AR transactivity of serum POP F1 extract alone, respectively. XERcomp and XARcomp: competitive receptor transactivity upon co-treatment with E2 or R1881, respectively. % agonistic XER and % XAR indicate a significant increase for the XER and XAR transactivity compared to the activity of the solvent control. % downregulated XER and % XAR indicated a significant decrease for the XER and XAR transactivity compared to the activity of the solvent control. % additive XERcomp and % additive XARcomp indicate a significant increase for the XERcomp and XARcomp transactivity compared to the activity of the E2-EC_40 _and R1881-EC_50 _solvent control, respectively. % antagonistic XERcomp and % antagonistic XARcomp indicate a significant decrease for the XERcomp and XARcomp transactivity compared to the activity of the E2-EC_40 _and R1881-EC_50 _solvent control, respectively. TEQ (TCDD equivalent): The TEQ value was calculated by interpolation of the AhR transactivity of serum extract to the TCDD dose-response curve using Sigmaplot program.

### Odd Ratios of correlation of serum POPs, metals and xenobiotic transactivities with the risk of breast cancer

Both before and after adjustment for the corresponding confounders, BC risk was associated with serum levels of PFOS (adjusted OR = 1.03, p = 0.05) and the sum of perfluorsulfonated acids (sumPFSA) (adjusted OR = 1.03, p = 0.02) (Table 4). Breast cancer risk was not associated with serum legacy POPs neither before nor after adjustment for the corresponding confounders (Table 4). However, the sum of legacy POPs and PFCs was significantly associated with the risk of BC (adjusted OR = 1.02, p = 0.01, Table 4).

**Table 4:** Odds ratios of breast cancer and 95% confidence intervals associated with PFCs and POPs among breast cancer patients and controls

	Raw^1^			Raw^2^			Adjusted^3^		
	
variables	n	OR	*p*	n	OR	*p*	n	OR	*p*
	
	(controls/cases)	(95% CI)		(controls/cases)	(95% CI)		(controls/cases)	(95% CI)	
**POPs**									

PFOS (ng/ml)	98/31	1.01 (1.003; 1.02)	**0.02**	69/9	1.01(0.99; 1.03)	0.23	69/9	1.03 (1.001; 1.07)	**0.05**

PFOA(ng/ml)	98/31	1.07 (0.88; 1.31)	0.50	69/7	0.94 (0.05; 1.38)	0.76	69/7	1.20 (0.77; 1.88)	0.43

sumPFSA(ng/ml)	98/31	1.013 (1.002;1.023)	**0.02**	69/15	1.01(0.99; 1.02)	0.37	69/15	1.03(1.00; 1.05)	**0.02**

sumPFCA(ng/ml)	98/31	1.05 (0.99; 1.11)	0.14	69/12	1.01(0.93; 1.10)	0.82	69/12	1.07(0.96; 1.18)	0.23

Sum PCB (μg/kg lipid)*	115/30	1.00(1.00; 1.00)	0.35	115/30	1.00(1.00; 1.00)	0.35	115/30	1.00(1.00; 1.00)	0.35

Sum OCP (μg/kg lipid)*	115/30	1.00(1.00; 1.00)	0.13	115/30	1.00(1.00; 1.00)	0.13	115/30	1.00(1.00; 1.00)	0.13

Sum PCB+sum OCP (μg/kg lipid)*	115/30	1.00(1.00; 1.00)	0.19	115/30	1.00(1.00; 1.00)	0.19	115/30	1.00(1.00; 1.00)	0.19

Sum PCB + sum OCP + sum PFSA + sum PFCA(ng/ml)	115/31	1.01(1.001; 1.014)	**0.03**	85/11	1.01(0.99; 1.02)	0.37	85/11	1.02(1.01; 1.04)	**0.01**

**Metals**									

Se (μg/kg)	115/30	1.0 (0.998; 1.001)	0.61	96/27	0.99(0.998; 1.001)	0.51	96/27	0.999(0.99; 1.001)	0.47

**Xenobiotic induced transactivities**									

XER(RLU/μg protein)	110/31	0.41(0.02; 7.50)	0.55	81/7	0.90 (0.001;663.8)	0.78	81/7	1.17(0.001; 1936)	0.97

*XER < 1*	23/8	28.9 (0.00; 301126)	0.48	11/3	9.49 (0.00;8.34e8)	0.81	11/3	0.00 (0.00; ∞)	0.65

*XER > 1*	87/23	0.09 (0.00; 23.8)	0.40	70/6	0.66(0.00; 9476)	0.93	70/6	1.85 (0.00; 79767)	0.91

XERcomp (RLU/μg protein)	110/31	2.15 (0.09; 53.77)	0.64	81/7	0.53(0.00; 18980)	0.91	81/7	0.22(0.00; 1166)	0.73

*XERcomp <1*	40/9	0.67 (0.00.00; 436)	0.90	20/1	0 (0.00; ∞)	0.995	20/1	0.00 (0.000; ∞)	0.99

*XERcomp >1*	70/22	0.60 (0.00; 1142)	0.89	61/6	0.06(0.00; 56092)	0.67	61/6	0.00(0.000; 111.6)	0.12

XAR(RLU/μg protein)	58/27	8.52 (1.55; 46.78)	**0.01**	49/11	28.5 (1.79; 452.6)	**0.02**	49/11	44.14(1.99; 975.7)	**0.02**

XARcomp(RLU/μg protein)	58/27	0.70(0.09; 5.31)	0.73	47/7	4.27 (0.17; 106.7)	0.38	47/7	5.04(0.20; 130.3)	0.33

AhR-TEQ(pg/g lipid)	113/29	0.99(0.99; 1.001)	0.09	83/6	1.003(0.98; 1.02)	0.77	83/6	1.01(0.98; 1.029)	0.58

Raw^1^: unadjusted data of full number of samples. Raw^2^: unadjusted data for the subset having all the corresponding adjustment variables (age, BMI, pregnancy, cotinine, breastfeeding, menopausal status), Adjusted^3^: fully adjusted data for the corresponding confounders. OR: odds ratio. CI: confidence interval. For sum PFSA, sum PFCA, sum PCB, sum OCP see legend of Table 2. For XER, XERcomp, XAR, XARcomp and TEQ see legend of Table 3. *: not adjusted since no confounder was identified.

The blood level of the trace element Se was not associated with BC risk (Table 4). None of the heavy metals were found to associate to the risk of breast cancer (data not shown). The integrated xenoestrogenic transactivity (XER) in serum was not associated with the risk of BC either before or after adjustment for confounders, whereas the integrated agonistic xenoandrogenic transactivity (XAR) in serum was significantly associated with the BC risk (adjusted OR = 44.1, p = 0.016, Table 4) both before and after adjusting for confounders. However, the AhR-TEQ was not found associated with the BC risk (Table 4).

## Discussion

This study aimed at evaluating the association between serum levels of legacy POPs and PFCs in Greenlandic Inuit breast cancer cases and their controls. The present data shows for the very first time a relation between the serum level of PFCs and the risk of breast cancer in Greenlandic Inuit. Moreover, the breast cancer cases also had a significant higher serum PCB level at the highest quartile. In addition, the cases elicited a higher frequency of subjects samples inducing POP related hormone-like agonistic xeno-ER (XER) and xeno-AR (XAR) transactivity being significantly different for XAR. In contrast, the serum POP related dioxin-like AhR-TEQ was lowest in BC cases, although the significance disappeared for the adjusted data.

Until now very few reports are published concerning the incidence of breast cancer in the Arctic population and to our knowledge none have ever evaluated the factors affecting the risk of breast cancer in Greenlandic Inuit. In the western world BC is the most common cancer for women [1,2], where established risk factors can explain less than 30% of the cases. The incidence of BC has traditionally been low among the Inuit, but since the 1970's a considerable increase has been observed [3,4] although still at a level being approximately 60% of the incidence in e.g. Denmark [58]. In the Arctic, before 1966 BC was reported absent from the western and central Canadian Arctic and from 1967 to 1980 only 2 BCs out of 107 cancers were found [59]. Breast cancer was studied over a 20-year period in Inuit populations in the circumpolar region. A total of 193 BC's were observed in women with an incidence increase from 28.2 per 100.000 in 1969-1973 to 34.3 per 100.000 in 1984-1988 [60]. In Greenland, the age adjusted incidence in women increased from 35 to 46.4/100000 in the period 1973-87 and 1988-97, respectively [58]. Among Greenlandic women, an increase is particular observed among older women e.g. at the age from 40 to 70 the increase was (~65/100,000) in 1973 - 87 compared to (~170/100,000) the period 1988-97. [58,4]. There is a pattern shift typically seen in low-risk countries with stagnation or falling rates after menopause to increased rates after menopause as observed in the Western countries. This pattern shift does not support a general improved diagnosis that would be expected to be reflected across all age groups. Ethnic and international differences in BC risk can mostly be explained by differences in environmental exposures and lifestyle, particularly reproductive and hormonal factors [6-8]. The increasing prevalence of obesity and type-2 diabetes [61], and change in breastfeeding from prolonged feeding continuing through childbearing years to a pattern of breastfeeding now more similar to the western world [4] could be consisting with the increase in Arctic BC rates. However, low parity, late age at first birth and obesity is also risk factors for hormone-related cancers such as uterine and ovarian cancer that have not elicited a parallel increase in incidence in the Arctic and thus other factors may be important for the increase in BC incidence [4].

In this study the median age and BMI were similar for BC cases and the controls and no difference in breastfeeding was found. The only established reproductive BC risk factor found significantly different from the controls was, that BC cases had a lower number of full term pregnancies, although this information was only obtained for approximately 50% and 77% of the cases and control participants, respectively. In contrast to the expected menopausal related BC risk a higher frequency of cases were premenopausal (55% vs. 18%), and for the controls a higher percent of the subjects were postmenopausal. We found that postmenopausal women had higher burden of PFCs and legacy POPs than premenopausal women both in cases and controls, supporting the known age-dependent bioaccumulations of these compounds [25,43]. It is expected that premenopausal women are at general lower risk for BC than postmenopausal women. However, we observed a higher frequency of premenopausal women in the case group during the sampling period 2000-2003. We suggest that the combined endogenous and xenobiotic related hormone disruption might be higher for cases compared to controls as hypothesized in the following: *Premenopausal cases*, although relatively shorter lifetime exposure to endogenous estrogens but higher actual estrogen levels, the higher legacy POPs and PFC serum levels contributes to a higher POP related xenobiotic estrogenic disruptions. For the *postmenopausal cases*, although lower actual level of endogenous estrogen, the BC risk is influenced by a longer lifetime estrogenic exposure and the higher legacy POPs and PFC serum level contributes to a higher POP related xenobiotic estrogenic disruptions. This is supported by our data analysis stratifying the menopause status showing that cases had higher levels of PFCs and legacy POPs than controls both for the premenopausal women as well as postmenopausal women. In addition, postmenopausal women might be at higher risk because of significant higher xenoandrogenic bioactivity in accordance to the hypothesis of Adams, J.B. [62].

Result from the questionnaire showed similar proportion of reported current smoking for cases and controls. However, since questionnaire is subjective, the measurement of serum cotinine is more precise to reflect the current smoking status. The lower level of serum cotinine for cases indicates that the cases smoked less but it can be a result of their disease although the non significant lower level of serum Cd in cases supports different smoking habits for cases.

The Arctic Inuit population have one of the highest burden of POPs globally, particularly in some districts in Greenland, and the hormone disrupting potential of the actual mixture of serum POPs have been reported [63]. The recent increase in BC incidence might be explained by the high burden of legacy POPs and increased exposure to new emerging POPs such as PFCs together with the recent transition in the Inuit diet from the traditional marine food to more western food and lifestyle factors such as smoking and alcohol intake.

The ubiquitous presence of PFAAs globally including the Arctic regions has been documented [64,25], and unlike legacy POPs the level of PFAAs in marine mammals from Greenland still show an increasing trend in the past years [65,66].

In the present study we measured 3 PFSA isomers and 7 PFCA isomers and the most abundant isomers were PFOS and PFOA, and to a less extend PFHxS. The statistic data for PFHxS was similar to that of PFOS (data not shown). Since PFOS and PFOA were the most abundant we use these two compounds as the representative isomer of PFSAs and PFCAs. For BC cases we observed a significant higher serum level of PFCs and for PCBs in the highest quartile, and the adjusted odds ratios (ORs) indicated a significant risk for BC in relation to the level of serum PFOS and sumPFSA and total sum of legacy POPs plus PFCs.

Our data shows to our knowledge for the very first time an association between the serum level of PFCs and the risk of breast cancer. The higher levels of PCBs in BC cases in the present study are supported by previous reported data [10-12,14].

In general, there have been reported non consistent association between serum fluorochemical levels and adverse health effects in human but elevated bladder cancer mortality among male workers exposed to PFOS for a minimum of one year [33]. In rats [38] and mice [39] studies upon gestational exposures to PFOA significant increase in mammary fibroadenomas and Leydig cell adenomas and significant reduction in mammary differentiation and gland development in dams and female offspring were reported, respectively. In addition, PFOA-exposed female pups showed stunted mammary gland epithelial branching and growth [39]. However, the hypothesis that the PPAR-α agonistic potential of PFOA was involved was not supported since PPAR-α null mice exhibited normal mammary gland development [39]. In contrast, over expression of PPAR-α during pregnancy in mice impair normal differentiation of the mammary gland [33]. Thus far, data suggest that PPAR-α is the most likely target of PFOA and PFOS with the former having highest affinity in both human and mouse isoforms [33]. Further research is needed to elucidate the role of the PPAR pathway on the mammary gland. Moreover, comparing animal (rodent) data with human health risk must always be done with some caution because the exposure profile and often the body elimination is very different: in animals the exposure is often with single compounds, higher concentration but relatively short time, whereas in human the exposure is lifelong with low doses and complex mixtures.

We found that the serum level of both PFAAs, PFOS and PFOA, as well as the legacy POPs were significant higher in the BC cases compared to the controls but the adjusted OR only indicated significant BC risk for PFOS and PFSA and upon pooling all the chemical groups. However, we found in both cases and controls a high inter-correlation between serum PFSA and PFCA (r = 0.85-0.96, p <0.05) and between these two PFC groups and the legacy POPs (r = 0.42-0.55, p <0.05). Taken that into consideration it cannot be predicted which single compounds or chemical group that play the main role in the observed increase in BC risk.

Preliminary data suggest that some PFCs might be weak xenoestrogens [41]. In adult rats it was found that PFOA led to a decrease in serum testosterone and increase in serum estradiol levels [33]. Future studies are needed to understand the possible roles of PFCs as endocrine disrupters on sex hormone homoeostasis and function.

In our study the total combined serum POP related xenobiotic bio-activities reflects the lipophilic legacy POPs only and does not include the more hydrophilic PFCs. In cases we found that the POP related serum agonistic xenoandrogenic transactivity was significantly associated with the risk of BC as well as a higher number of subjects with significant increased serum agonistic xenoestrogenic transactivity.

With reference to the induced XAR a significant association to BC risk was found both before and after adjustment for related confounders. Using a similar analytical system the biological effects of the total combined xenoestrogens have been set in relation to the risk of BC by N. Olea and his co-workers [67,68]. It is well established that exposure to estrogens can increase the risk of breast cancer mainly attributed via their potential to increase the receptor induced cell proliferation and thereby expand possible gene damage and mutations in e.g. either tumour suppressing genes or proto-oncogenes causing inactivation or activation of the gene expressions of growth factors that act on mammary epithelial cells in an autocrine or paracrine loop [69]. Thus the body burden of exogenous estrogens such as POP related xenostrogens may further increase the BC risk.

Adams, J.B. [62] proposed a role of adrenal androgens (AA) in the aetiology of breast cancer. Premenopausal women that develop BC tend to have subnormal serum levels of AA and thus lower androgens acting via the AR opposing the estrogenic stimulated cell growth during this life period. In contrast, subjects developing the disease postmenopausal have supranormal levels of AA, and the elevated AA levels stimulate cell growth by the action of 5-androsten-3β, 17β-diol, termed hermaphrodiol, via its combination with the ER in a milieu having low concentrations of the classical E2. It might be hypothesized that the significantly higher combined serum POP related xenoAR activity level in cases has contributed to the BC development predominantly in the postmenopausal cases whereas the higher frequency of cases eliciting significant agonistic POP related xenoestrogenic transactivity might affect the BC risk in both pre- and postmenopausal cases. However, further research is needed before any conclusion can be taken.

In the present study we observed a significant lower level of AhR-TEQ in BC cases compared to their controls although the significance disappeared upon adjustment for the corresponding confounders (age, BMI, pregnancy, breastfeeding and cotinine). The higher serum sum POP/sumPCB levels in cases may explain the observed difference between cases and controls. Non-dioxin-like PCBs are shown to have the potential to antagonize the AhR pathway [70,71], supporting our unpublished *in vitro *studies (manuscript in preparation). Moreover, we found in an earlier study that Inuit with high serum levels of PCB had lower AhR-TEQ compared to Europeans with lower PCB levels [55]. Dioxins are reported to have antiestrogenic potentials [72-74]. It can be speculated whether the lower dioxin-like AhR-TEQ level in cases could play a role in BC risk via its lower antiestrogenic potential. Alternatively, it may be explained by differences between cases and controls regarding metabolic pathways involved in the biotransformation of both mono-ortho PCBs and estrogens as suggested by Demers et al. 2002 [75].

In spite of the POPs the traditional diet might also offer some protection against breast cancer because the marine diet is rich in 22:6, n-3 fatty acids and the antioxidant selenium (Se) [76], both factors suggested to have inhibitory effect on breast cancer [77,78] and chemically induced carcinogenesis in animals [79]. In addition, the intake of n-3 fatty acids is associated to the level of Vitamin A and D. An inverse relation between Zn and breast cancer was reported [80], and solar radiation and thus Vitamin D was suggested to reduce BC risk [81]. The intake of Zn has decreased since 1976 [48,82]. Thus, by changing to the western-lifestyle food intake the Greenlandic Inuit have a decreased intake of food factors, which might be protective against the risk of BC, including n-3 fatty acids, Zn, Vitamin D and Se.

In this study we found a trend to lower n-3/n-6 fatty acid ratio and lower Se but significantly higher levels of serum Zn in BC cases. To evaluate whether these factors contributed to the development of BC needs further research. However, a recent physiologically based pharmacokinetic (PBPK) model accounting for any given physiologic lifetime history using data on pregnancies, height, weight, and age, estimated the values of physiologic parameters (e.g., organ volume, composition, and blood flow) throughout a woman's entire life was developed. This PBPK model showed the limitations of using a single sample value obtained around the time of diagnosis for lifetime exposure assessment and point out the need to estimate the past POP exposure during time windows that are hypothesized to be mechanistically critical in carcinogenesis [83]. These factors may to some degree explain the controversial reports on POP exposure and breast cancer risk.

In the present study the metals were measured in the whole blood which primarily reflects the actual metal exposure. The limitation in reflecting the body burden prior to the disease will be further discussed in a manuscript particularly focusing on the effects of metals on the risk of breast cancer from the same study population (manuscript is in preparation).

There are some weaknesses in the presented study. Firstly, the few subjects involved, 31 cases and 115 controls, gives a poor statistically power. However, the highly related serum PFC levels with the risk of BC cancer did persist in all our effort to make up a better case-control frequency match. The number of controls in the West region (especially in Nuuk) was higher than other regions and may have impact on the final results. Therefore we also evaluated the data by reducing the number of Nuuk control subjects by matching the age and BMI with the cases in Nuuk to obtain similar cases/control ratio as for the other regions. Similar data was found between the data presented and upon including the reduced and matched data of Nuuk controls (not shown). However, a trend of higher level of organochlorine pesticides (OCPs) and competitive xenoestrogenic transactivity (XERcomp) for cases was observed being significant by including the reduced and matched data of Nuuk controls, whereas the observed significant difference between cases and controls at the highest PCB quartile and XAR transactivity disappeared probably due to reduced statistical power (data not shown).

## Conclusions

The presented data show a higher level of PFCs and legacy POP in BC cases indicating that the level of serum POPs in particularly PFCs might be risk factors in the development of breast cancer in Greenlandic Inuit. Furthermore, our data suggest that the higher level of legacy POP related xenoestrogenic and xenoandrogenic agonistic activities in cases compared to controls can contribute to the development of BC in Inuit. Further investigations are needed to document these study conclusions.

## Abbreviations

AA: adrenal androgens; AR: androgen receptor; AhR: aryl hydrocarbon receptor; AMAP: Arctic Monitoring and Assessment Programme; BC: Breast cancer; BMI: Body Mass Index; p,p'-DDT: dichlorodiphenyltrichloroethane; p,p'-DDE: p,p'-dichlorodiphenyl-dichloroethylene; E2:estradiol; ER: estrogen receptor; ESI: electrospray ionization; HCB: hexachlorobenze; β-HCH: β-hexachlorocyclohexane; LC-MS-MS: liquid chromatography-tandem mass spectrometry; OCPs: organochlorine pesticides; ORs: odds ratios; PBPK: physiologically based pharmacokinetic; PCBs: polychlorinated biphenyls; PCDD/PCDF: Polychlorinated Dibenzodioxins and Polychlorinated Dibenzofurans; PFAAs: perfluoroalkyl acids; PFCs: Perfluorinated contaminants; PFCAs: perfluorocarboxylated acids; PFDA: perfluorodecanoic acid; PFDoA: perfluorododecanoic acid; PFHpA: Perfluoroheptanoic acid; PFHxS: perfluorohexane sulfonate; PFNA: perfluorononanoic acid; PFOA: perfluorooctanoic acid; PFOS: perfluorooctane sulfonate; PFOSA: perfluorooctane sulfonamide;PFSAs: perfluorosulfonated acids;PFTrA: perfluorotridecanoic acid; PFUnA: perfluoroundecanoic acid;POPs: persistent organic pollutants;SPE-HPLC: Solid Phase Extraction - High-Performance Liquid Chromatography;TEQ: TCDD equivalents;XAR: xeno-androgen receptor transactivity of serum alone;XER: xeno-estrogen receptor transactivity of serum alone;XARcomp: competitive xenoandrogenic transactivity;XERcomp: competitive xenoestrogenic transactivity.

## Conflict of interest statement

The authors declare that they have no competing interests.

## Authors' contributions

ECB-J conceived the study and made the overall study design, participated in data analyses and drafted the manuscript; ML participated in the study design, performed the statistical analyses, participated in drafting the manuscript and reviewed the manuscript drafts; RB were responsible for the PFC analyses and reviewed the manuscript drafts, PA were responsible for the AhR-CALUX analyses and reviewed the manuscript drafts; GA performed the trace element analyses and reviewed the manuscript drafts, TK performed the AR-transactivity analyses, guided the ER- transactivity analyses and reviewed the manuscript drafts, MG performed the SPE-HPLC analyses and reviewed the manuscript drafts, GM was the responsible person for cases and control sampling in Greenland, PK and PN took the samples from cases and reviewed the manuscript drafts, ED participated in the study design, handed over the control samples from Nuuk and reviewed the manuscript drafts. All authors read and approved the final manuscript.

## Supplementary Material

Additional file 1 **Results after being stratified by menopausal status**. Levels of POPs, metals and POP related xenobiotic induced receptor transactivities of BC cases and controls within the premenopausal and postmenopausal women are given. Click here for file
