# Supplementary material for: Perfluorinated compounds are related to breast cancer risk in greenlandic inuit: A case control study
Source: Environ Health. 2011 Oct 6;10:88. doi: 10.1186/1476-069X-10-88 (PMC3203030; doi:10.1186/1476-069X-10-88)
Supplement: Additional file 1 — Results after being stratified by menopausal status. Levels of POPs, metals and POP related xenobiotic induced receptor transactivities of BC cases and controls within the premenopausal and postmenopausal women are given. [file 1476-069X-10-88-S1.DOCX]

Additional Table 1A. Results after stratified by menopausal status

|  | **Premenopausal** | | | | | | | | | |
| --- | --- | --- | --- | --- | --- | --- | --- | --- | --- | --- |
|  | case | | | |  | control | | | | P value |
|  | n | median | 95% CI | Min-max |  | n | median | 95% CI | Min-max |  |
| **POPs** |  |  |  |  |  |  |  |  |  |  |
| PFOS(ng/ml) | 11 | 37.5 | 22.8;57.9 | 11.6-96.6 |  | 15 | 20.1 | 17.0;49.0 | 9.4-87.5 | 0.26 |
| PFOA(ng/ml) | 11 | 2.4 | 1.4;2.7 | 0.2-3.5 |  | 15 | 2.2 | 1.7;4.0 | 0.7-7.6 | 0.33 |
| Sum PFSA(ng/ml) | 11 | 40.6 | 25.0;62.6 | 13.2-104.5 |  | 15 | 24.0 | 19.0;52.7 | 10.0-92.2 | 0.26 |
| Sum PFCA(ng/ml) | 11 | 5.4 | 4.2;11.4 | 3.3-18.3 |  | 15 | 5.2 | 3.8;8.3 | 2.0-14.8 | 0.31 |
| Sum PCB(µg/kg lipid) | 11 | 857.1 | 614.8;2586 | 150.6-4529 |  | 16 | 1546 | 1143;2228 | 450.9-4888 | 0.29 |
| *>2645(µg/kg lipid)* | *3* | *8801* | *4527;14584* | *8017-11849* |  | *1* | *4889* |  |  | - |
| SumOCP(µg/kg lipid) | 11 | 1650 | 1104;4111 | 250.4-7320 |  | 16 | 2009 | 1472;2516 | 364.4-4594 | 0.83 |
| SumDL-PCB(µg/kg lipid) | 11 | 111.8 | 44.3;255.7 | 17.9-527.2 |  | 16 | 172.5 | 128.3;227.6 | 55.1-439.9 | 0.09 |
| SumPCB+sumOCP (µg/kg lipid) | 11 | 2758 | 1747;6668 | 400.9-11848 |  | 16 | 3512 | 2633;4727 | 815.3-9483 | 0.74 |
| SumPCB+sumOCP+sumPFSA+sumPFCA (ng/ml) | 11 | 66.2 | 38.0;120.0 | 22.1-231.8 |  | 16 | 58.2 | 49.3;85.5 | 30.7-133.4 | 0.77 |
| **Metals** |  |  |  |  |  |  |  |  |  |  |
| Se(µg/kg lipid) | 10 | 240.0 | 33.4;791.4 | 115.7-1877 |  | 16 | 198.4 | 163.0;363.2 | 120.5-855.8 | 0.46 |
| Cd(µg/kg lipid) | 10 | 0.9 | 0.5;1.4 | 0.13-1.91 |  | 16 | 1.0 | 0.9;1.7 | 0.3-2.9 | 0.17 |
| Hg(µg/kg lipid) | 10 | 18.4 | 9.1;35.1 | 1.5-61.7 |  | 16 | 8.0 | 0.1;54.9 | 0.9-194.1 | 0.37 |
| Pb(µg/kg lipid) | 10 | 27.4 | 18.4;46.1 | 10.8-69.9 |  | 16 | 50.2 | 39.8;69.1 | 19.4-112.7 | **0.02** |
| Zn(µg/kg lipid) | 10 | 6204 | 5233;7299 | 4250-8583 |  | 16 | 4938 | 4598;5420 | 3908-7166 | **0.02** |
| **POP related xenobiotic induced receptor transactivities** |  |  |  |  |  |  |  |  |  |  |
| XER(RLU/µg protein) | 11 | 1.07 | 1.05;1.19 | 1.0-1.3 |  | 16 | 1.06 | 1.03;1.16 | 0.9-1.3 | 0.56 |
| *% agonistic XER* |  | *54.5%* |  |  |  |  | *37.5%* |  |  | 0.38 |
| *% downregulated XER* |  | 0 |  |  |  |  | 0 |  |  |  |
| XERcomp(RLU/µg protein) | 11 | 1.05 | 0.96;1.10 | 0.9-1.2 |  | 16 | 1.06 | 1.01;1.06 | 0.9-1.3 | 0.38 |
| *% additive XERcomp* |  | *18.2%* |  |  |  |  | *37.5%* |  |  | 0.28 |
| *% antagonistic XERcomp* |  | *9.1%* |  |  |  |  | *0* |  |  | 0.22 |
| XAR(RLU/µg protein) | 9 | 1.03 | 0.85;1.25 | 0.6-1.6 |  | 10 | 1.08 | 0.86;1.27 | 0.7-1.5 | 0.86 |
| *% agonistic XAR* |  | *10.0%* |  |  |  |  | *10.0%* |  |  | 1.0 |
| *% downregulated XAR* |  | *20.0%* |  |  |  |  | *11.1%* |  |  | 0.60 |
| XARcomp (RLU/µg protein) | 9 | 0.70 | 0.58;0.94 | 0.4-1.2 |  | 10 | 0.64 | 0.53;0.88 | 0.3-1.0 | 0.64 |
| *% additive XARcomp* |  | *0* |  |  |  |  | *-* |  |  |  |
| *% antagonistic XARcomp* |  | *40.0%* |  |  |  |  | *55.6%* |  |  | 0.50 |
| AhR-TEQ (pg/l) | 9 | 161.6 | 1.3;648.4 | 31.4-1411 |  | 14 | 369.8 | 275.6;457.3 | 123.1-739.1 | 0.10 |
| AhR-TEQ(pg/g lipid) | 9 | 30.5 | 11.8;81.0 | 9.0-153.3 |  | 14 | 48.9 | 35.1;63.1 | 11.5-113.7 | 0.34 |

Additional Table 1B. Results after stratified by menopausal status

|  | **Postmenopausal** | | | | | | | | | |
| --- | --- | --- | --- | --- | --- | --- | --- | --- | --- | --- |
|  | case | | | |  | control | | | | P value |
|  | n | median | 95% CI | Min-max |  | n | median | 95% CI | Min-max |  |
| **POPs** |  |  |  |  |  |  |  |  |  |  |
| PFOS(ng/ml) | 9 | 74.0 | 52.0;95.3 | 35.5-124 |  | 57 | 40.9 | 39.2;60.3 | 3.9-172.0 | **0.001** |
| PFOA(ng/ml) | 9 | 3.1 | 2.4;5.2 | 1.5-7.2 |  | 57 | 3.0 | 2.7;3.7 | 0.6-6.8 | 0.16 |
| Sum PFSA(ng/ml) | 9 | 80.2 | 56.2;103.2 | 38.1-132.7 |  | 57 | 43.4 | 42.2;64.8 | 5.0-183.7 | **0.001** |
| Sum PFCA(ng/ml) | 9 | 14.4 | 8.7;17.5 | 5.4-21.4 |  | 57 | 8.4 | 8.2;12.2 | 1.5-28.1 | **0.04** |
| Sum PCB(µg/kg lipid) | 9 | 2222 | 1355;4248 | 332-6528 |  | 73 | 2281 | 2170;2678 | 692.8-5640 | 0.91 |
| *>2645(µg/kg lipid)* | *3* | *9673* | *5672;14029* | *8265-11615* |  | *26* | *6872* | *6839;8619* | *5033-13299* | **0.02** |
| SumOCP(µg/kg lipid) | 9 | 3512 | 2014;4476 | 379.1-5425 |  | 73 | 2806 | 2574;3246 | 927.1 | 0.94 |
| SumDL-PCB(µg/kg lipid) | 9 | 211.9 | 120.6;325.6 | 33.0-425.7 |  | 73 | 256.2 | 243.5;297.1 | 94.7-596.0 | 0.27 |
| SumPCB+sumOCP (µg/kg lipid) | 9 | 5985 | 3457;8636 | 711.2-11615 |  | 73 | 5141 | 4768;5899 | 1657-13330 | 0.98 |
| SumPCB+sumOCP+sumPFSA+sumPFCA (ng/ml) | 9 | 137.7 | 99.1;168.9 | 57.1-198.1 |  | 73 | 73.3 | 79.6;107.7 | 18.8-297.5 | **0.005** |
| **Metals** |  |  |  |  |  |  |  |  |  |  |
| Se(µg/kg lipid) | 9 | 200.2 | 136.1;450.4 | 101.0-697.3 |  | 73 | 304.8 | 345.2;504.3 | 90.6-1805 | 0.16 |
| Cd(µg/kg lipid) | 9 | 0.8 | 0.2;1.6 | 0.0-2.7 |  | 73 | 1.2 | 1.2;1.8 | 0.0-6.5 | 0.35 |
| Hg(µg/kg lipid) | 9 | 17.2 | 12.2;24.9 | 9.7-36.1 |  | 73 | 15.4 | 14.9;21.7 | 0.4-81.2 | 0.38 |
| Pb(µg/kg lipid) | 9 | 71.6 | 42.3;93.9 | 16.7-129.2 |  | 73 | 78.8 | 79.7;115.4 | 16.6-498.6 | 0.24 |
| Zn(µg/kg lipid) | 9 | 5393 | 4376;6231 | 3013-7487 |  | 73 | 4825 | 4707;5357 | 2431-10823 | 0.51 |
| **POP related xenobiotic induced receptor transactivities** |  |  |  |  |  |  |  |  |  |  |
| XER(RLU/µg protein) | 9 | 1.06 | 0.9;1.1 | 0.9-1.2 |  | 69 | 1.12 | 1.08;1.13 | 0.6-1.3 | **0.04** |
| % agonistic XER |  | *33.3%* |  |  |  |  | *36.2%* |  |  | 0.86 |
| % downregulated XER |  | *22.2%* |  |  |  |  | *2.9%* |  |  | **0.01** |
| XERcomp(RLU/µg protein) | 9 | 1.06 | 0.9;1.1 | 0.8-1.1 |  | 69 | 1.06 | 1.05;1.09 | 0.9-1.2 | 0.27 |
| *% additive XERcomp* |  | *11.1%* |  |  |  |  | *18.8%* |  |  | 0.57 |
| *% antagonistic XERcomp* |  | *22.2%* |  |  |  |  | *2.9%* |  |  | **0.001** |
| XAR(RLU/µg protein) | 7 | 0.96 | 0.78;1.39 | 0.7-1.6 |  | 40 | 0.90 | 0.81;0.94 | 0.4-1.2 | **0.06** |
| *% agonistic XAR* |  | *28.6%* |  |  |  |  | *2.4%* |  |  | **0.008** |
| *% downregulated XAR* |  | *0* |  |  |  |  | *33.3%* |  |  | 0.07 |
| XARcomp (RLU/µg protein) | 7 | 0.67 | 0.52;1.03 | 0.6-1.3 |  | 40 | 0.67 | 0.64;0.78 | 0.3-1.2 | 0.47 |
| *% additive XARcomp* |  | *14.3%* |  |  |  |  | *0* |  |  | **0.02** |
| *% antagonistic XARcomp* |  | *71.4%* |  |  |  |  | *41.0%* |  |  | 0.14 |
| AhR-TEQ (pg/l) | 9 | 436.1 | 230.3;724.5 | 116.9-996.6 |  | 73 | 474.4 | 496.5;667.4 | 111.9-1583 | 0.24 |
| AhR-TEQ(pg/g lipid) | 9 | 56.8 | 25.8;118.5 | 11.4-188.0 |  | 73 | 58.6 | 59.9;78.5 | 20.0-196.2 | 0.52 |

Additional Table 2. Comparison of exposure variables between pre-menopausal and post-menopausal women

|  | **case** | | | | |  | **control** | | | |  |
| --- | --- | --- | --- | --- | --- | --- | --- | --- | --- | --- | --- |
|  | premenopause | | postmenopause | | p value^1^ |  | premenopause | | postmenopause | | p value^2^ |
|  | n | median | n | median |  |  | n | median | n | median |  |
| **Age** | 11 | 45 | 9 | 56 | **0.002** |  | 15 | 51.5 | 57 | 59 | **<0.0001** |
| **POPs** |  |  |  |  |  |  |  |  |  |  |  |
| PFOS(ng/ml) | 11 | 37.5 | 9 | 74.0 | **0.01** |  | 15 | 20.1 | 57 | 40.9 | 0.20 |
| PFOA(ng/ml) | 11 | 2.4 | 9 | 3.1 | **0.04** |  | 15 | 2.2 | 57 | 3.0 | 0.56 |
| Sum PFSA(ng/ml) | 11 | 40.6 | 9 | 80.2 | **0.01** |  | 15 | 24.0 | 57 | 43.4 | 0.22 |
| Sum PFCA(ng/ml) | 11 | 5.4 | 9 | 14.4 | **0.03** |  | 15 | 5.2 | 57 | 8.4 | 0.08 |
| Sum PCB(µg/kg lipid) | 11 | 857.1 | 9 | 2222 | 0.12 |  | 16 | 1546 | 73 | 2281 | **0.003** |
| *>2645(µg/kg lipid)* | *3* | *8801* | *3* | *9673* | *0.25* |  | *1* | *4889* | *26* | *6872* | **-** |
| SumOCP(µg/kg lipid) | 11 | 1650 | 9 | 3512 | 0.36 |  | 16 | 2009 | 73 | 2806 | **0.004** |
| SumDL-PCB(µg/kg lipid) | 11 | 111.8 | 9 | 211.9 | 0.14 |  | 16 | 172.5 | 73 | 256.2 | **0.001** |
| SumPCB+sumOCP (µg/kg lipid) | 11 | 2758 | 9 | 5985 | 0.22 |  | 16 | 3512 | 73 | 5141 | **0.002** |
| SumPCB+sumOCP+sumPFSA+sumPFCA (ng/ml) | 11 | 66.2 | 9 | 137.7 | **0.01** |  | 16 | 58.2 | 73 | 73.3 | 0.17 |
| **Metals** |  |  |  |  |  |  |  |  |  |  |  |
| Se(µg/kg lipid) | 10 | 240.0 | 9 | 200.2 | 0.72 |  | 16 | 198.4 | 73 | 304.8 | **0.03** |
| Cd(µg/kg lipid) | 10 | 0.9 | 7 | 0.8 | 0.89 |  | 16 | 1.0 | 70 | 1.2 | 0.94 |
| Hg(µg/kg lipid) | 10 | 18.4 | 9 | 17.2 | 0.72 |  | 16 | 8.0 | 73 | 15.4 | 0.29 |
| Pb(µg/kg lipid) | 10 | 27.4 | 9 | 71.6 | **0.01** |  | 16 | 50.2 | 73 | 78.8 | **0.01** |
| Zn(µg/kg lipid) | 10 | 6204 | 9 | 5393 | 0.15 |  | 16 | 4938 | 73 | 4825 | 0.78 |
| **POP related xenobiotic induced receptor transactivities** |  |  |  |  |  |  |  |  |  |  |  |
| XER(RLU/µg protein) | 11 | 1.07 | 9 | 1.06 | 0.40 |  | 16 | 1.06 | 69 | 1.12 | 0.81 |
| *% agonistic XER* |  | *54.5%* |  | *33.3%* | *0.34* |  |  | *37.5%* |  | *36.2%* | *0.92* |
| *% downregulated XER* |  | *0* |  | *22.2%* | *0.10* |  |  | *0* |  | *2.9%* | *0.49* |
| XERcomp(RLU/µg protein) | 11 | 1.05 | 9 | 1.06 | 0.87 |  | 16 | 1.06 | 69 | 1.06 | 0.92 |
| *% additive XERcomp* |  | 18.2% |  | 11.1% | 0.66 |  |  | *37.5%* |  | *18.8%* | 0.11 |
| *% antagonistic XERcomp* |  | 9.1% |  | 22.2% | 0.34 |  |  | *0* |  | *2.9%* | 0.49 |
| XAR(RLU/µg protein) | 10 | 1.03 | 7 | 0.96 | 0.82 |  | 9 | 1.08 | 40 | 0.90 | **0.04** |
| *% agonistic XAR* |  | 10.0% |  | 28.6% | 0.32 |  |  | *10.0%* |  | *2.4%* | 0.27 |
| *% downregulated XAR* |  | 20.0% |  | 0 | 0.21 |  |  | *11.1%* |  | *33.3%* | 0.19 |
| XARcomp (RLU/µg protein) | 10 | 0.70 | 7 | 0.67 | 0.85 |  | 9 | 0.64 | 40 | 0.67 | 0.92 |
| *% additive XARcomp* |  | *0* |  | *14.3%* | *0.22* |  |  | *0* |  | *0* | - |
| *% antagonistic XARcomp* |  | *40.0%* |  | *71.4%* | *0.20* |  |  | *55.6%* |  | *41.0%* | 0.43 |
| AhR-TEQ (pg/l) | 9 | 161.6 | 9 | 436.1 | 0.15 |  | 14 | 369.8 | 73 | 474.4 | **0.03** |
| AhR-TEQ(pg/g lipid) | 9 | 30.5 | 9 | 56.8 | 0.29 |  | 14 | 48.9 | 73 | 58.6 | 0.06 |

p value^1^: premenopause vs. postmenopause within the case group, p value^2^: premenopause vs. postmenopause within the control group.
